# Caseload per Year in Robotic-Assisted Minimally Invasive Esophagectomy: A Narrative Review

**DOI:** 10.3390/cancers16203538

**Published:** 2024-10-19

**Authors:** Ibrahim Büdeyri, Nader El-Sourani, Ann-Kathrin Eichelmann, Jennifer Merten, Mazen A. Juratli, Andreas Pascher, Jens P. Hoelzen

**Affiliations:** Department of General, Visceral and Transplant Surgery, University Hospital Muenster, University of Muenster, Albert-Schweitzer-Campus 1, 48149 Muenster, Germany; ibrahim.buedeyri@ukmuenster.de (I.B.);

**Keywords:** RAMIE, robotics, esophagectomy, caseload per year, failure to rescue, surgical training

## Abstract

**Simple Summary:**

Esophageal surgery is associated with a high hospital mortality. Research shows that hospitals performing more esophageal surgeries have better outcomes due to a higher likelihood of rescuing patients from complications. Since 2004, Germany has mandated a minimum required caseload per year for hospitals to ensure quality care and, as of 2023, increased the annually required number of complex esophageal operations from 10 to 26. This review will explore how the caseload per year impacts the quality of patient care and surgical training, especially regarding robotic-assisted minimally invasive esophagectomy (RAMIE), which promises greater precision and better outcomes for esophageal cancer surgeries.

**Abstract:**

Esophageal surgery is deemed one of the most complex visceral operations. There is a well-documented correlation between higher caseload and better outcomes, with hospitals that perform more surgeries experiencing significantly lower mortality rates. The approach to caseload per year varies across different countries within Europe. Germany increased the minimum annual required caseload of complex esophageal surgeries from 10 to 26 starting in 2023. Furthermore, the new regulations present challenges for surgical training and staff recruitment, risking the further fragmentation of training programs. Enhanced regional cooperation is proposed as a solution to ensure comprehensive training. This review explores the benefits of robotic-assisted minimally invasive esophagectomy (RAMIE) in improving surgical precision and patient outcomes and aims to evaluate how the caseload per year influences the quality of patient care and the efficacy of surgical training, especially with the integration of advanced robotic techniques.

## 1. Introduction

Esophageal surgery in Germany has a high hospital mortality rate in international comparison; the mortality rates of 8.8–12.6% are the highest in visceral surgery [1]. For many procedures, there is a correlation between the number of cases and the quality of treatment, i.e., a volume–outcome relationship, which is strong for esophageal surgery [2]. Hospitals with a median of 62 cases per year in Germany between 2010 and 2015 had a halved hospital mortality rate compared to hospitals with a median of two cases annually [3]. This is primarily attributed to failure to rescue (FTR) in hospitals with a smaller caseload, as complications are not rarer in hospitals with higher caseloads, but patients with complications are less likely to die there [3]. Moreover, there was a survival advantage for patients who were treated in the certified cancer centers [4].

Caseload regulations (i.e., 10 cases per year) for esophageal surgery were introduced in Germany in 2004. Hospitals must demonstrate annually in a forecasting procedure that they will achieve the caseload in the following year in order to be entitled to remuneration [5]. A total of 3697 complex esophageal procedures were performed in 2018 at 327 German hospitals [6]. To combat the issue of hospitals with a low caseload performing complex surgeries, the Federal Joint Committee increased the required caseload per year for complex esophageal operations from 10 to 26 in 2023, thus decreasing the number of hospitals from 327 down to 111 in 2024 [7,8]. According to simulations, travel times to hospitals for esophageal surgery are only extended by an average of 11 min (31 min in total) across Germany due to the increased caseload per year [6]. However, patients already travel on average 22–24 min further than necessary and skip the next hospital providing care [9]. A long distance to treatment can be associated with reduced survival in cancer patients [10]. At the same time, visits by relatives, which are important for the psychological support of people undergoing surgery, can be more difficult [11].

An important issue is the impact of caseload per year on surgical training [12,13]. The restrictions associated with caseload per year also make it more difficult to recruit junior surgeons, who need consistent exposure to a variety of core procedures such as those of hernia, gallbladder, and diverticulitis. Consequently, this dynamic can lead to a fragmentation of core visceral surgery training programs. Close regional cooperation is a possible solution [12,13,14]. In order for good cooperation to become established, it must be structurally promoted. Good cooperation could enable further surgical training within rotation plans.

**Figure 1:**
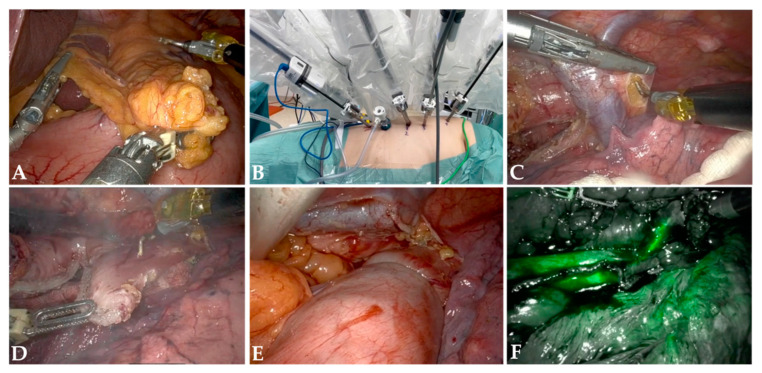
(**A**) Gastric mobilization. (**B**) Thoracic trocar placement. (**C**) Division of azygos vein. (**D**) Esophageal transection. (**E**) Esophagogastrostomy. (**F**) Indocyanine green (ICG) fluorescence guided assessment of anastomotic perfusion.

Robotic-assisted minimally invasive esophagectomy (RAMIE) has emerged as a cutting-edge approach in the surgical management of esophageal cancer [15]. Leveraging advanced robotic technology, RAMIE offers multiple clinical benefits that enhance surgical precision and improve patient outcomes [16]. One of the primary advantages of RAMIE is the enhanced surgical precision provided by robotic systems. These systems offer surgeons three-dimensional visualization and improved dexterity, which are critical for the complex anatomy of the esophagus. The most critical steps of the RAMIE procedure are summarized in Figure 1.

The aim of this review is to address whether a defined caseload per year for RAMIE has an impact on the quality of patient care and the surgical training program.

## 2. Rationale for RAMIE

The use of robotic systems in esophagectomy significantly improves the accuracy of tumor resections while minimizing damage to the surrounding tissues. This precision is particularly important in the esophagus, where delicate structures such as the thoracic aorta and the vagus nerve are in close proximity. The high-definition, three-dimensional imaging provided by robotic systems significantly improves the accuracy of tissue dissection as it offers up to 10× magnification, allowing surgeons to see structures more clearly and making it easier to identify and preserve critical anatomical features. The enhanced visualization helps to reduce the risk of inadvertent injury to the surrounding tissues [17]. By seeing the surgical field in greater detail, surgeons can make more informed decisions, leading to more precise and effective surgeries [18,19]. Another notable advantage of RAMIE is the use of advanced instrumentation that enhances surgical precision in dissection and reconstruction in complex esophageal surgeries by allowing for multidimensional rotation and articulation at angles not possible with standard laparoscopic instruments [20,21]. This increased dexterity is particularly beneficial in esophagectomy, where accessing and manipulating tissues in a confined space can be challenging. The ergonomic benefits for surgeons performing RAMIE cannot be overlooked. Conventional esophagectomy procedures can be physically demanding, leading to surgeon fatigue and potentially impacting surgical outcomes. Robotic systems, however, allow for more comfortable and stable operating positions [22]. This reduction in surgeon fatigue can enhance focus during long and complex procedures [23].

The clinical benefits of RAMIE extend to patient outcomes as well [24]. The precision of robotic instruments and improved visualization typically result in lower intraoperative blood loss and reduced postoperative pain. Hoelzen et al. reported that RAMIE is associated with less postoperative pain, improving patient comfort, and facilitating earlier mobilization [25]. This quicker recovery is a significant advantage, as it can lead to shorter hospital stays and a faster return to normal activities. Manigrasso et al. highlighted that patients undergoing RAMIE experience fewer wound infections and pneumonia, which can significantly impact recovery, contributing to better overall patient outcomes [21]. Tagkalos et al. demonstrated that robotic-assisted procedures result in fewer respiratory infections and a decreased incidence of anastomotic leaks, which are significant complications that can impact patient recovery and survival rates [26]. The precision of robotic surgery allows for more meticulous dissection and suturing, reducing the likelihood of such complications. This reduction in postoperative complications is a critical factor in improving patient outcomes and reducing the overall burden on healthcare systems. Siaw-Acheampong et al. have shown that RAMIE leads to shorter stays in the intensive care unit and lower rates of pulmonary complications, such as pneumonia [27]. A summary of existing evidence regarding the impact of RAMIE on postoperative complications is shown in Table 1.

The superiority of RAMIE over conventional esophagectomy is supported by Level 1 evidence available in the literature [43]. The ROBOT trial compared RAMIE to open transthoracic esophagectomy (OTE), demonstrating that RAMIE led to fewer overall surgery-related and cardiopulmonary complications, less postoperative pain, better short-term postoperative functional recovery, and improved quality of life [44]. Harriott et al., Banks et al., Esagian et al., and Perry et al. demonstrated in their meta-analyses that patients undergoing RAMIE have better overall outcomes and lower mortality rates compared to those undergoing traditional open esophagectomy [45,46,47,48]. The ability to offer a less invasive yet highly precise surgical option expands the potential for successful treatment of esophageal cancer in a broader patient population.

**Figure 2:**
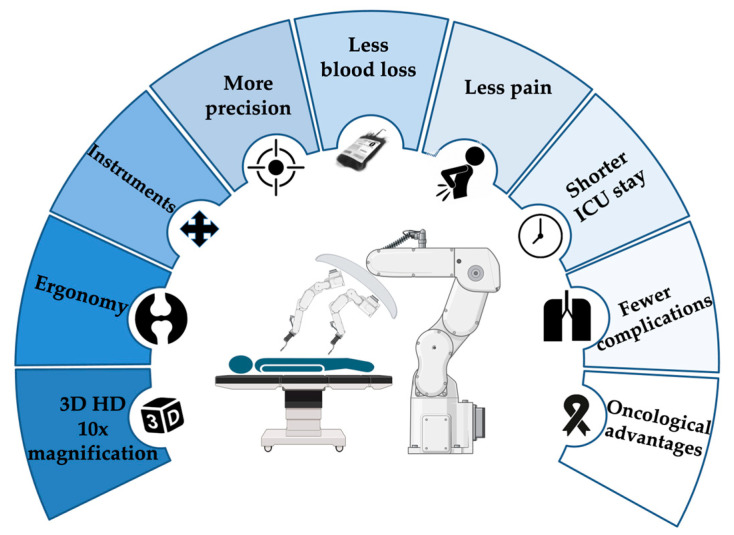
Rationale for RAMIE. This figure was created in BioRender.com.

Precise dissection and clear margins are crucial in cancer surgery to ensure complete removal of the tumor, increase the yield of the harvested lymph nodes, and minimize the risk of recurrence [49]. This not only helps in thorough tumor resection but also in preserving vital structures, which is essential for maintaining the patient’s quality of life post-surgery. There is a growing body of evidence that minimally invasive surgery allows patients to return earlier to intended oncologic treatment postoperatively [50,51]. Recently, it has been highlighted in a multicentric randomized controlled trial that robotic pancreatectomy promoted a faster return to adjuvant therapy compared to open surgery [52]. Similarly, it could be hypothesized that RAMIE may facilitate an earlier initiation of adjuvant therapy. Faster recovery times and fewer complications mean that patients are in better overall health to tolerate additional treatments such as chemotherapy or radiation therapy, which are often necessary adjuncts in the comprehensive management of esophageal cancer. The rationale for RAMIE is summarized in Figure 2.

## 3. Caseload Requirements Across Europe

Setting a minimum caseload per year for esophagectomy procedures is a strategy employed by health systems to ensure high-quality care and improved outcomes. The approach to caseload per year varies across different countries within Europe, with each country setting its own thresholds based on evidence emerging from local studies [53,54,55,56,57,58,59,60,61,62]. Different countries have varying minimum case load requirements due to several factors, including differences in healthcare systems, hospital density, and political as well as economic considerations. For instance, Germany has a decentralized healthcare system with a relatively high number of hospitals spread across the country, whereas countries like the Netherlands [63] and those in Scandinavia [64] have more centralized, specialized centers. This centralization often leads to higher minimum caseloads in these countries due to the concentration of expertise and resources. Economic factors also play a role, as larger centers can benefit from economies of scale, making them more cost-efficient. Smaller hospitals in decentralized systems, on the other hand, may face pressure to justify higher minimum caseloads due to limited resources and the need to maintain surgical proficiency.

In Germany, the required caseload per year for esophagectomy procedures has seen significant adjustments over recent years. As of 2023, hospitals in Germany must now perform at least 26 esophagectomies per year to retain the status of a certified national esophageal center [7]. The rationale behind this regulatory change is to concentrate these high-risk operations in hospitals with sufficient experience and expertise, which enables healthcare teams, including physicians and nurses, to detect complications at an earlier stage. High-volume centers also have the necessary infrastructure and protocols to manage severe complications promptly and effectively [3,9]. Studies, such as those by Hue et al. and Fuchs et al., underscore the importance of surgical volume in achieving better outcomes for patients undergoing esophagectomy [56,65]. Higher surgical volumes are consistently associated with lower complication rates and higher survival rates, reflecting the impact of experience and proficiency on surgical outcomes. By raising the minimum required caseload per year, German healthcare policymakers aim to enhance patient safety and optimize healthcare resources by ensuring that esophagectomy procedures are performed in centers with proven competence.

Kjaer et al. indicates that reorganizing the care for esophageal cancer patients in Denmark has significantly lowered the 30-day mortality rate from 10.7% in the 1990s [57] to 1.7% in 2013 [59]. Additionally, centralizing treatment in specialized centers, along with an increase in esophageal cancer cases, has nearly doubled the number of surgeries per center, though survival rates have not improved during the study period. This centralization, initiated by the National Health Service, began in 2003, reducing the number of departments treating esophageal cancer from 26 to 4 by 2006 [59]. Denmark’s hospital volumes for these surgeries were higher than any other country, with most esophagectomies conducted in hospitals performing over 40 procedures annually [55].

In England, the treatment of esophago-gastric cancer was centralized from 113 centers to 34, so that the median number of cases in the centers rose from 21 to 55. During the same period, the 30-day mortality rate fell from 7.4% to 2.5% [61].

Hospitals in the Netherlands are required to perform a minimum of 20 esophagectomies annually to maintain certification [53,60,62]. Studies from the Netherlands, including those by Ketel et al. [58], have consistently shown that concentrating esophagectomy procedures in specialized centers leads to improved patient outcomes, including lower rates of postoperative complications and higher long-term survival rates. The Dutch healthcare system’s emphasis on high surgical volumes reflects a commitment to optimizing patient outcomes through specialization and expertise [54].

## 4. Quality and Complication Management

RAMIE represents a significant advancement in the surgical management of esophageal cancer. Ensuring high-quality outcomes in RAMIE involves meticulous quality management, effective complication management, robust process quality, and comprehensive risk stratification. National and international standards for the minimum required caseload per year in RAMIE vary but the underlying principle remains consistent, i.e., concentrating complex surgical procedures in high-volume centers improves patient outcomes. Lang et al. discussed the importance of establishing and adhering to these standards to ensure high-quality care [12]. By aligning with these standards, healthcare systems can optimize resource allocation, enhance surgical proficiency, and improve overall patient outcomes.

This chapter explores these critical aspects, integrating insights from recent studies and highlighting best practices in the field.

### 4.1. Quality Management

Quality management in RAMIE encompasses certification, continuous training, process monitoring, and adherence to evidence-based protocols. These elements are critical for maintaining high standards of care. Hospitals performing RAMIE must obtain certification from recognized health authorities. Accreditation ensures that these institutions adhere to stringent standards and guidelines for surgical care. Certification programs often require hospitals to demonstrate their capability through caseload, outcome data, and compliance with best practices. To implement the most up-to-date standards, it is essential to contribute to the existing body of evidence by actively participating in research. This process helps maintain high-quality outcomes and patient safety. Continuous monitoring of surgical processes and outcomes is essential for quality assurance. Regular audits and reviews of surgical procedures help identify areas for improvement. Metrics such as operation time, blood loss, complication rate, and patient recovery time are crucial indicators that should be closely monitored. Implementing robust monitoring systems allows hospitals to identify trends, address issues promptly, and maintain high standards of care [17]. Implementing and following standardized surgical protocols helps maintain consistency and safety across procedures. These protocols should be based on the latest evidence and best practices in robotic surgery.

**Figure 3:**
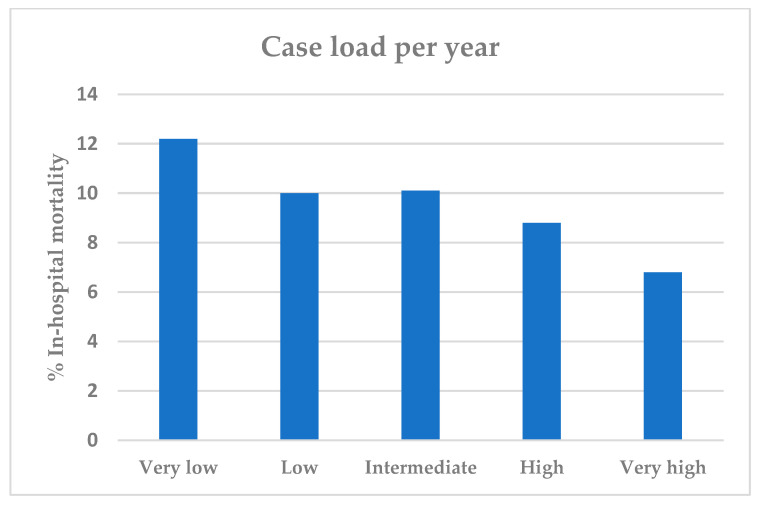
In-hospital mortality after complex esophageal interventions between 2010 and 2015, stratified by caseload quintile. Median caseload per year is divided into 5 categories: very low 2, low 10, intermediate 15, high 26 and very high 62 cases per year. Figure adapted from Nimptsch et al. 2018 [3].

The volume–outcome relationship in RAMIE suggests that higher procedural volumes are associated with better patient outcomes. Di et al. conducted a systematic review and meta-analysis demonstrating that hospitals with higher volumes of esophagectomy procedures had lower in-hospital mortality rates and improved overall survival [66]. In accordance with that, Nimptsch et al. analyzed the in-hospital mortality in relation to the annual caseload of complex esophageal surgeries in German hospitals from the years 2010 to 2015 and found out that low mortality rates can be achieved if the patients are treated in hospitals with a high caseload, as shown in Figure 3 [3]. This relationship underscores the importance of centralizing esophagectomy procedures in high-volume centers to enhance patient safety and optimize outcomes.

Defining a minimum case load per center, rather than per surgeon, reflects the belief that surgical outcomes depend not only on the individual surgeon’s performance but also on the institutional infrastructure and the multidisciplinary team’s ability to manage complications. However, focusing on individual caseloads for surgeons could be a valid consideration in ensuring surgical proficiency. Birkmeyer et al. investigated the relationship between surgeon volume and operative mortality in eight major surgical procedures in the United States [67]. The study found that higher surgeon volume is significantly associated with lower operative mortality rates, independent of hospital volume, particularly in complex procedures including esophagectomy and pancreatic resection. The authors conclude that surgical outcomes could improve if patients undergoing high-risk procedures are directed to surgeons with higher case volumes. A single surgeon performing a higher volume of cases may gain more direct experience and technical refinement than two surgeons splitting cases, but outcomes are not solely based on volume. Aspects like the ability to manage complications, the quality of postoperative care, and the experience of the entire surgical team are critical to patient outcomes. In this context, an experienced center can ensure high-quality care, regardless of the number of surgeons involved. The correlation between hospital volume and surgical quality has been well documented. Nimptsch et al. found that hospitals performing higher volumes of esophagectomy procedures had lower FTR rates and in-hospital mortality [3], highlighting the critical role of experience in managing complex surgeries [68]. This evidence supports the implementation of a minimum required caseload per year as a quality assurance measure in RAMIE.

### 4.2. Complication Management

Effective complication management is a cornerstone of successful RAMIE and involves several key strategies such as increasing the minimum required caseload per year and implementing FTR concepts. FTR refers to the inability to prevent a patient from dying after a complication occurs. A higher minimum required caseload per year encourages hospitals to develop more refined risk stratification protocols, namely standard operating procedures (SOP). High-volume centers typically follow SOPs for early detection and management of complications, reducing FTR rates and enhancing overall patient safety [3,69]. The preoperative planning and intraoperative monitoring are essential components in identifying risks early and taking preventive measures. Implementing effective FTR strategies is critical for improving patient outcomes and reducing mortality rates. Utilizing advanced imaging technologies and precise surgical techniques enables early detection and prevention of potential complications. Managing complications requires a coordinated effort from an interdisciplinary team, including surgeons, gastroenterologists, anesthesiologists, intensivists, radiologists, and nursing staff. A collaborative approach ensures that any complications are promptly and effectively addressed. This teamwork is critical for providing comprehensive care and improving patient outcomes, as complications are not rarer in high-volume centers, but thanks to FTR strategies those complications are less likely to end with mortality [3]. Close monitoring of patients during the postoperative period is crucial for early identification and management of complications such as anastomotic leaks, respiratory issues, and infections. Preoperative endoscopic diagnostics and early endoscopic management of complications are just as important. In case of an anastomotic insufficiency, endoscopic vacuum therapy has been proven to be beneficial in preventing fatal complications [70,71]. It is not uncommon in high-volume centers for the thoracic aorta to be stented prophylactically by vascular surgeons or interventional radiologists if there is an anastomotic leakage and a higher risk of aortic erosion bleeding [72]. Larger hospitals with more interdisciplinary experience managing complications may better prevent morbidity from progressing to mortality.

## 5. Influence of Caseload on Surgical Performance, Training, and Learning Curves in RAMIE

The volume of the caseload performed by a surgical team significantly influences their performance and outcomes [9,73]. This is especially true in the context of RAMIE, where the complexity of the procedure necessitates a high level of skill and proficiency. This chapter explores how case volume impacts surgical performance and the learning curves associated with RAMIE, integrating insights from recent studies.

High case volumes are associated with better surgical performance due to increased experience and familiarity with the procedure. Surgeons and surgical teams at high-volume centers develop greater proficiency and are better able to manage intraoperative challenges and complexities. Higher case volumes lead to improved technical skills, reduced operation times, and lower complication rates [21,26]. The correlation between high case volumes and improved outcomes underscores the importance of centralizing complex surgical procedures in specialized centers. Surgeons performing RAMIE at high-volume centers can achieve proficiency more quickly due to the frequent practice and reinforcement of skills. This reduced learning curve duration translates into safer and more effective surgeries, with lower rates of intraoperative complications. Various parameters such as surgical time, complication rates, and postoperative outcomes are used to assess the progression of the learning curve for minimally invasive esophagectomies. However, no universally validated or accepted outcome measures currently exist [74]. Further research is needed to identify the optimal parameters that will ensure the best patient outcomes and determine the appropriate duration of proctoring. To ensure that the learning curve for RAMIE is safe for patients, several strategies can be implemented. These strategies focus on structured training, continuous education, mentorship, simulation training, progressive case complexity, and regular performance assessments.

Continuous professional development is vital in the field of robotic surgery. Surgeons and their teams must regularly participate in advanced training programs to stay updated with the latest techniques and technologies [75]. Ongoing training helps surgeons refine their skills and stay current with advancements in the field. The adoption of more complex and innovative procedures necessitates the development of more efficient and safe implementation programs to minimize morbidity related to the learning process. Standardized training programs have been shown to be effective in improving outcomes for surgical procedures [76]. Having experienced surgeons proctor and mentor less experienced surgeons during the initial phase of their learning curve can ensure patient safety. This guidance helps in the early identification and correction of technical errors and provides immediate feedback to improve performance. Proctoring and mentorship are essential components of safe and effective surgical training [17,77].

Utilizing simulation and virtual reality training allows surgeons to practice and refine their skills without risking patient safety. These technologies provide realistic scenarios that can help build confidence and competence in performing RAMIE. The use of advanced simulation technologies is critical for enhancing surgical training and ensuring patient safety [26,78]. Surgeons should start with less complex cases and gradually progress to more challenging ones as they become more proficient. This stepwise approach ensures that they build their skills and confidence progressively. A gradual approach to case complexity is essential for ensuring safe and effective surgical training.

Regular assessment of surgical performance and continuous feedback from peers and mentors are crucial for maintaining high standards. These evaluations help identify areas for improvement and reinforce good practices. Regular performance evaluations help surgeons refine their skills and enhance patient outcomes. A structured training pathway is essential for implementing RAMIE, shortening the learning curve and making RAMIE safer and more effective. Müller et al. analyzed a training pathway built on proficiency-based progression in RAMIE [79]. This approach involves setting specific performance benchmarks that surgeons must achieve before advancing to more complex cases. The study found that proficiency-based progression ensures that surgeons acquire the necessary skills at each stage of their training, reducing the risk of complications and improving surgical outcomes. Pickering et al. described in a systematic review and meta-analysis the existing evidence on learning curves in RAMIE and underscored the importance of standardization [80]. Van der Sluis et al. examined the learning curve for RAMIE based on 312 procedures and highlighted that as surgeons gained experience, there were marked improvements in operative time, blood loss, and patient outcomes [81]. The initial learning phase for thoracic laparoscopic RAMIE included 70 procedures conducted over 55 months, but with structured proctoring, the number of cases and time needed to reach proficiency was significantly reduced [81]. Hernandez et al. investigated the learning curve for robotic-assisted esophagogastrectomy by analyzing outcomes from 45 cases and found that surgeons typically required around 20 cases to reach proficiency, with notable improvements in operative time, blood loss, and postoperative outcomes beyond this point [82].

Last but not least, a crucial issue is the impact of the annual caseload on surgical training. Limited case volumes can hinder the development of surgical skills, particularly for trainees who need consistent exposure to a variety of procedures. The restrictions associated with a lower caseload also make it challenging to attract and retain staff. Consequently, this dynamic can exacerbate existing staff shortages in smaller centers and lead to a fragmentation of core visceral surgery training programs. Close regional cooperation between hospitals and surgical departments offers a potential solution to these challenges. For such collaborations to be effective, they need to be supported by structured programs and institutional incentives. Regional partnerships could facilitate shared training opportunities, allowing surgeons to rotate between different centers and gain comprehensive experience through exposure to complex cases. This rotational model would not only enhance training in specialized procedures but also ensure a more balanced distribution of surgical expertise across regions. By pooling resources, hospitals can provide a broader range of surgical cases for trainees, promoting well-rounded skill development. Moreover, it may encourage experienced surgeons to mentor junior colleagues across different sites, enriching the training environment. Such cooperative efforts could help mitigate staff shortages by creating more attractive career pathways that combine the benefits of both high-volume and smaller centers. Ultimately, a structured training curriculum within the regional cooperation and mentorship programs could play a critical role in maintaining high standards of surgical expertise and patient safety.

## 6. Conclusions

The influence of case volume on surgical performance and learning curves in RAMIE is significant, with higher volumes leading to better outcomes due to increased experience and proficiency. Structured training pathways, continuous professional development, mentorship, simulation training, progressive case complexity, and regular performance assessments can enhance the learning curve for RAMIE. These strategies ensure high-quality care and improved patient outcome.

## 7. Future Directions

Future research should evaluate the long-term impact of these standards on patient outcomes, healthcare costs, and system efficiency. Advancements in surgical techniques and technologies, including RAMIE, may influence future requirements for caseloads. As healthcare systems evolve, establishing and refining minimum caseload standards for esophagectomy procedures will be crucial for quality improvement.

## Figures and Tables

**Table 1:** Impact of RAMIE on postoperative complications compared to MIE. RN injury: recurrent nerve injury. MIE: minimally invasive esophagectomy.

Author	Year	Country	Complications (%, RAMIE vs. MIE)			
			Pulmonary	RN Injury	Anastomotic Leak	Chyle Leak
Suda [28]	2012	Japan	6 vs. 20	38 vs. 75	38 vs. 10	0 vs. 10
Weksler [29]	2012	USA	9.1 vs. 15	9.1 vs. 3.8	9.1 vs. 15	NA
Park [30]	2016	Korea	13 vs. 24	8.1 vs. 2.3	NA	7.6 vs. 1.6
He [31]	2018	China	19 vs. 7.4	15 vs. 11	11 vs. 3.7	0 vs. 3.7
Chao [32]	2018	Taiwan	5.9 vs. 18	21 vs. 29	0 vs. 5.9	NA
Deng [33]	2019	China	9.6 vs. 7.7	14 vs. 7.7	5.8 vs. 3.8	0 vs. 1.9
Tagkalos [34]	2019	Germany	12 vs. 18	NA	12 vs. 18	NA
Zhang [35]	2019	China	6.1 vs. 7.6	6.1 vs. 4.5	7.6 vs. 4.5	0 vs. 1.5
Chen [36]	2019	China	15 vs. 24	13 vs. 32	9.3 vs. 3.7	1.9 vs. 3.7
Motoyama [37]	2019	Japan	0 vs. 0	24 vs. 47	5 vs. 8	5 vs. 3
Yang [38]	2019	China	8.9 vs. 13	29 vs. 15	12 vs. 14	1.5 vs. 0.7
Harbison [39]	2019	USA	11 vs. 19	NA	14 vs. 15	3 vs. 2.2
Babic [40]	2021	Germany	8.5 vs. 8.5	NA	7.9 vs. 11	NA
Tsunoda [41]	2021	Japan	18 vs. 44	7 vs. 20	18 vs. 18	NA
Kingma [24]	2022	The Netherlands, Germany, Taiwan, USA, France, Hong Kong, Brazil, UK, Italy	25 vs. 33	2 vs. 6	20 vs. 22	5 vs. 6
Hoelzen [42]	2023	Germany	7.6 vs. 20.6	NA	14.2 vs. 22.2	NA
